# Nanodevices Tend to Be Round

**DOI:** 10.3390/mi12030330

**Published:** 2021-03-20

**Authors:** Georges Pananakakis, Gérard Ghibaudo, Sorin Cristoloveanu

**Affiliations:** IMEP-LAHC, Université Grenoble Alpes, Minatec/INPG, 3 Parvis L. Neel, CS 50257, CEDEX 1, 38016 Grenoble, France; georges.pananas@orange.fr (G.P.); gerard.ghibaudo@grenoble-inp.fr (G.G.)

**Keywords:** nanoelectronics, nanowire, junctionless, MOSFET, gate-all-around, SOI

## Abstract

Under several circumstances, a nanowire transistor with a square cross-section behaves as a circular. Taking the Gate-All-Around junctionless transistor as a primary example, we investigate the transition of the conductive region from square to circle-like. In this case, the metamorphosis is accentuated by smaller size, lower doping, and higher gate voltage. After defining the geometrical criterion for square-to-circle shift, simulation results are used to document the main consequences. This transition occurs naturally in nanowires thinner than 50 nm. The results are rather universal, and supportive evidence is gathered from inversion-mode Gate-All-Around (GAA) MOSFETs as well as from thermal diffusion process.

## 1. Introduction

Most vertical nanowires (NW) have circular cross-sections. Even planar nanowires tend to become round, albeit the lithography process is expected to produce rectangular shapes. After oxidation and/or chemical etching, a square piece of semiconductor eventually ends up being round. In both Gate-All-Around (GAA [1,2]) field-effect transistor (FET) and Four-Gate FET (G^4^-FET [3]), the current filament starts to form in the middle of the structure and has initially a circular shape. Round current filaments are also observed during transistor breakdown or operation of resistive memory (RRAM). Furthermore, the photon beam in a square fiber is circular. Similar observation is for a tiny filet of falling water from a square pipe.

There are many other examples suggesting that Mother Nature prefers small things to be round. On the technical side, we can argue that the mechanisms leading to rounded forms are governed by more or less similar second-order differential equations, where the boundary conditions tend to suppress the corner effects.

**Figure 1:**
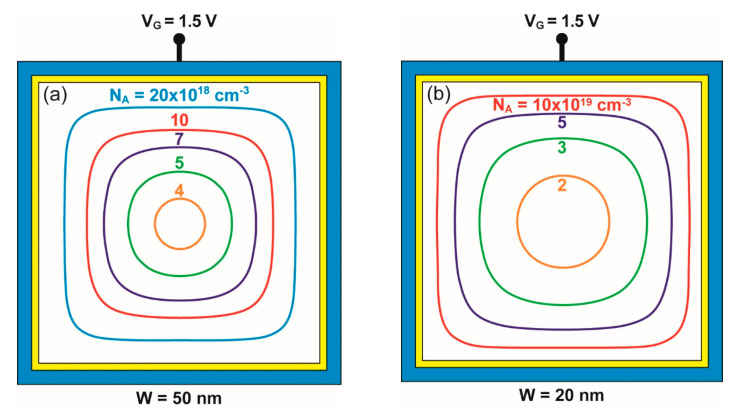
Contours of the neutral region in (**a**) 50 nm wide and (**b**) 20 nm wide nanowires with variable body doping and a square cross-section. As doping decreases, the large square-shape neutral region transforms into a smaller size region with a circular shape. Gate bias V_G_ = 1.5 V.

In this paper, the conditions enabling the intriguing transition from squared to circular forms are investigated. We focus on GAA square nanowires with high body doping (Figure 1). This device is also named junctionless FET because, in principle, there is no need for overdoping the source and drain terminals [4]. The surrounding gate controls the extent of the depletion region—in other words, the area of the neutral section of the nanowire where the current flows. The ON state corresponds to no depletion, and the OFF state occurs as soon as the body of the nanowire becomes fully depleted and the current is suppressed.

Figure 1 shows that the neutral region turns from a ‘large’ square into a ‘small’ circle. This motivates our interest for exploring the parameters of the metamorphosis from square to circular nanowires. A preliminary question addressed in Section 2 is: When can we affirm that a form with central symmetry looks more similar to a circle than a square?

Section 3 contains a systematic discussion of the critical dimension, doping, and gate bias for such a transition to occur. Section 4 shows briefly how our methodology can be generalized to other examples taken from the nanowire processing and operation.

## 2. From Square to Circle

We consider p-channel depletion-mode GAA MOSFETs. A typical device features a square cross-section with size W between 100 nm down to 10 nm. The concentration of acceptor dopant (N_A_ ≈ 10^18^–10^20^ cm^−3^) is selected such as to ensure a high current level while avoiding full depletion at low gate voltage. The gate dielectric (SiO_2_) is 2 nm thick and does not contain fixed charges or traps. The transistor is ‘long’, meaning that the fringing fields from source and drain terminals do not cause short-channel and 3D effects [5]. Numerical simulations were performed with a home-made 2D solver of the Poisson equation. Quantum carrier confinement and subband splitting are irrelevant for nanowires larger than 10 nm.

As soon as the gate bias V_G_ exceeds the flatband voltage, the depletion region forms at the surface and gradually becomes wider. Beyond the threshold voltage (V_G_ ≥ V_th_), an electron inversion layer starts growing at the Si–SiO_2_ interface, and the depletion region stops expanding.

The transition from the neutral region to the depletion region is not abrupt and occurs over a distance defined by the Debye length L_D_. An empirical expression for the one-dimensional (1D) profile of hole concentration in a single-gate planar device is [6]

(1) $$p\left(x\right)=\frac{N_{A}}{2}\left[1+\mathrm{th}\left(\frac{x-W_{d}}{{\alpha \;L}_{D}}\right)\right]$$

where x is the distance from the gate oxide interface (located at x = 0), W_d_ is the depletion depth, and α ≈ 1.7 (adjusted by calibrating Equation (1) with numerical computations). Conventional relations for Debye and depletion length are

(2) $$L_{D}=\sqrt{\frac{\varepsilon_{s}\mathrm{kT}}{q^{2}N_{A}}}\;W_{d}=\sqrt{\frac{2\varepsilon_{s}\psi_{s}}{{q\;N}_{A}}}$$

where ε_s_ is Si permittivity and Ψ_s_ surface potential. 

Following Equation (1), the depletion depth corresponds to the position where the hole concentration reaches half of the doping level p(x = W_d_) = N_A_/2. Due to the collaboration of multiple gates, W_d_ can be higher than the theoretical 1D value in Equation (2) [6], but the criterion above to determine it remains valid.

Figure 1a illustrates the behavior of a medium-size nanowire (W = 50 nm). The contours in Figure 1a show the maximum depletion region (or the minimum neutral region) achieved for a high enough gate voltage. The lower the doping, the more circular the neutral region is, until full depletion is achieved. Similar contours are observed in Figure 1b for a thinner NW (W = 20 nm) with higher doping. A parallel to the shrinking of the neutral region is the fog that dissolves by warming a square window to leave a small circular dot.

**Figure 2:**
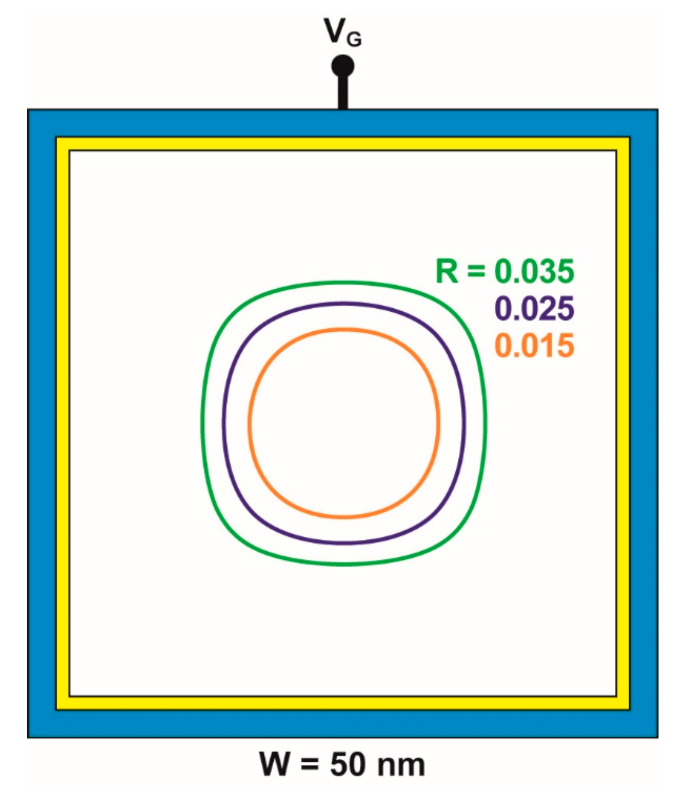
Contours of the neutral region selected in the vicinity of the square–circle transition defined for a form factor R = 0.025. For R = 0.035, the shape is slightly square-like, whereas for R = 0.015, it is more circle-like. W = 50 nm, V_G_ = 1.5 V, N_A_ doping range from 4.8 to 6.6 × 10^18^ cm^−3^.

The choice of the contour that better represents the transition from square to circle is challenging. Parameters based on area and perimeter are not sensitive enough; for example, the area-to-perimeter ratio is the same (W/4) for both extreme cases (square and circle). A more suitable choice is the distance d from the center of the nanowire to the points located on the contour. The ratio R between the standard deviation σ_d_ and the average distance <d> varies from 0 (perfect circle) to 0.1 (perfect square). For the contours of Figure 1a, the form factor is R = 0.003 for N_A_ = 4 × 10^18^ cm^−3^ (quasi-circle) and R = 0.082 for N_A_ = 2 × 10^19^ cm^−3^ (square-like). We have selected visually R = 0.025 as a criterion to represent the square-to-circle transition. Figure 2 shows contours with 0.015 ≤ R ≤ 0.035 that illustrate the transition region. The choice of an alternative criterion will not modify quantitatively the results discussed in the following.

## 3. Impact of Nanowire Size, Doping, and Bias

**Figure 3:**
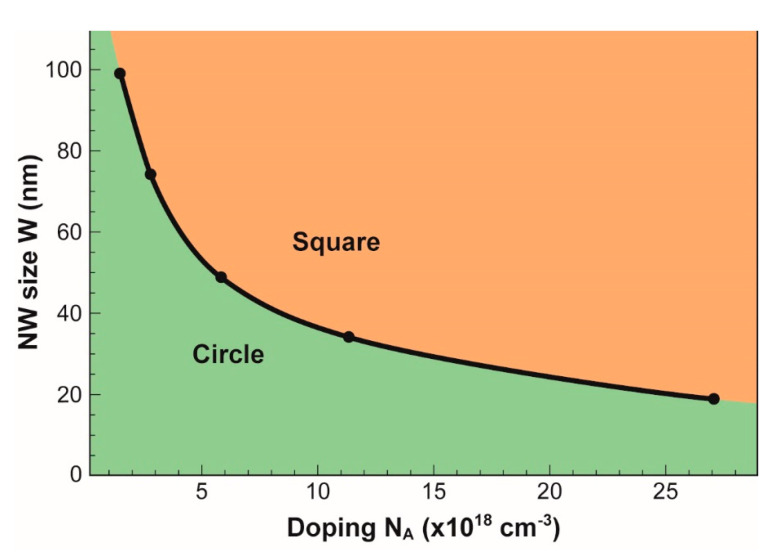
Frontier between square and circular shapes of the neutral region as a function of nanowire doping and size. The boundary corresponds to R = 0.025 criterion. V_G_ = 1.5 V.

The boundary between square-like and circle-like shapes of the minimum neutral region is calculated with the criterion above, for different combinations of nanowire doping and size. Figure 3 shows that a wide NW (W ≥ 100 nm) behaves naturally as a square, unless doping is light (N_A_ < 10^18^ cm^−3^). In contrast, nanowires smaller than 25 nm are either fully depleted (i.e., no neutral region) or circle-like. In medium-size NWs, the neutral region tends to be circular, except for heavy doping (N_A_ > 10^19^ cm^−3^ for W = 40 nm).

**Figure 4:**
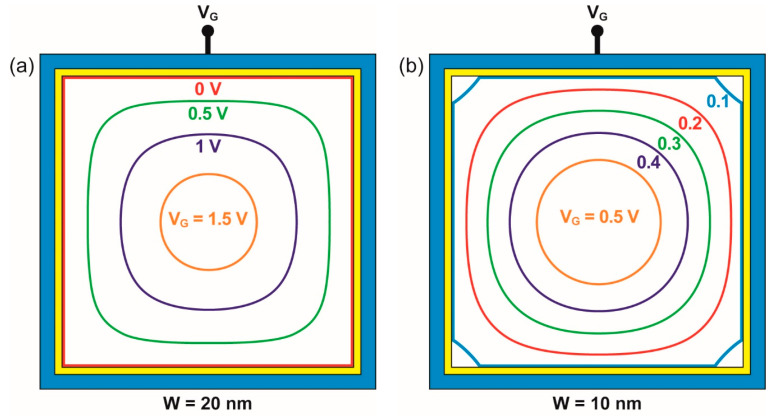
Contours of the neutral region in (**a**) 20 nm wide and (**b**) 10 nm wide nanowires for variable gate bias. As V_G_ increases, the large square-shape neutral region transforms into a smaller-size region with circular shape. Doping concentration N_A_ = 2 × 10^19^ cm^−^^3^.

For the given dimensions and doping of the physical square NW, the conductive filament can be made circular by gate action. Figure 4 reproduces the expansion of the depletion region with gate voltage in two heavily doped and small nanowires. At flat-band condition (i.e., V_G_ = 0), the doping concentration is constant in the whole cross-section of the square NW. For positive gate bias V_G_, the device starts to be depleted. The depletion region emerges from the surface and follows the body contour, preserving a square shape at low bias (V_G_ < 1 V in Figure 4a). For increased V_G_, the combination of the vertical and horizontal components of the electric field results in a higher effective field at the corners than at the mid-gate. The depletion region expands faster from the corners, leading to a clear rounding of the neutral region.

**Figure 5:**
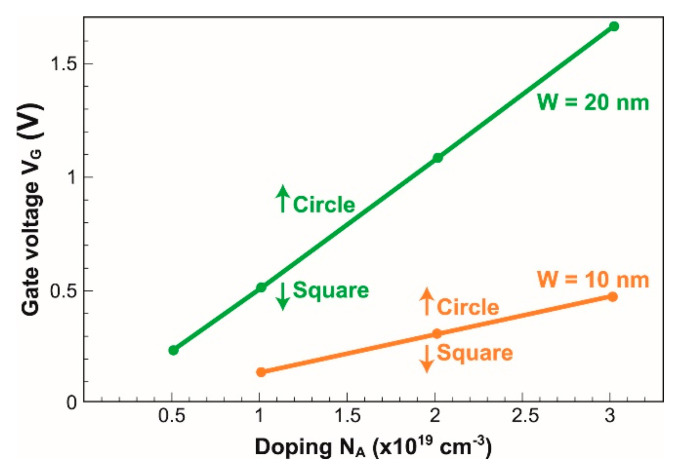
The lines indicate the frontier between square and circular shapes as a function of gate bias and nominal doping level. The conductive cross-section is circular above each line and square-like below.

The gradual change of the neutral region size and shape, from square to circle, is clearly visible in a 20 nm large NW (Figure 4a). Only at high gate voltage (V_G_ = 1.5 V) does the neutral region become circular. In a very thin NW (10 nm, Figure 4b), the conductive region is always circular except at very low bias (V_G_ < 0.3 V). The results are summarized in Figure 5 that shows the square-to-circle transition in V_G_–N_A_ space for two nanowires. The conducting NWs tend to be circular above each curve and square underneath. A higher gate voltage and/or a lower doping expand the surrounding depletion region and reinforce the circular aspect of the neutral (conductive) section.

The key point in all cases—whatever the original size, doping, and gate bias—is that the conductive filament becomes circular when the ‘diameter’ reaches about 10 nm. This empirical rule motivates our study. We now focus on the carrier concentration in the NWs.

**Figure 6:**
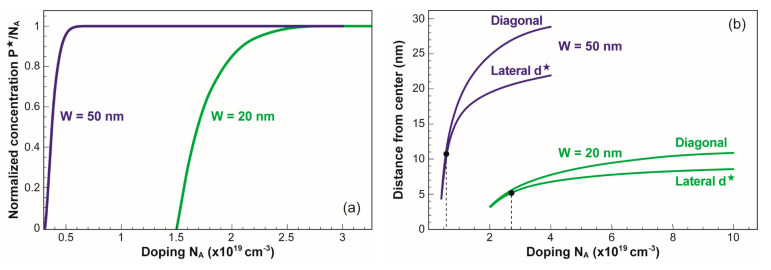

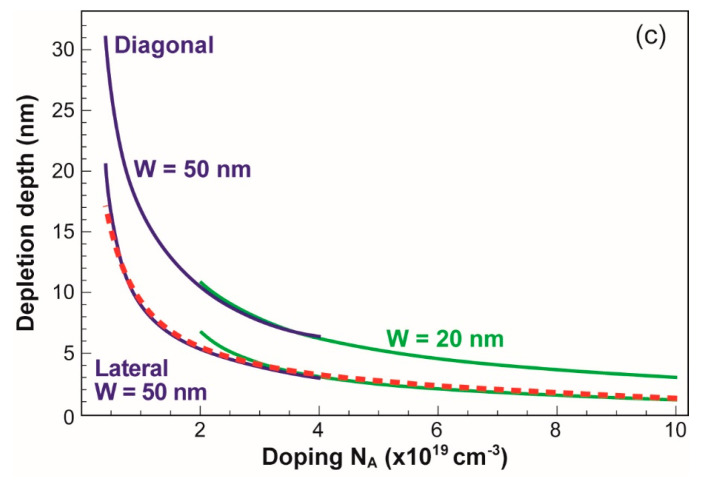
(**a**) Normalized hole concentration at the NW center versus nominal doping. (**b**) Minimum distance from the center to the edge of the conducting region measured in horizontal and diagonal directions. (**c**) Depletion depth, measured laterally from the middle of the NW edge or diagonally from the corner, versus doping. The dotted line indicates the results obtained with Equation (1). V_G_ = 1.5 V.

Figure 6a shows the concentration of holes at the center of the NW normalized by the nominal doping. It varies from zero (full depletion) at relatively low doping to N_A_ (neutral core). The smaller the NW, the slower the variation. The minimum distance d* from the center to the periphery of the conducting section increases from zero (full depletion) to W/2 in flat-band condition, as shown in Figure 6b. The bullets indicate the doping corresponding to the square-to-circle transition; for lower doping, when the circular shape is achieved, d* is obviously the radius.

Reciprocally in Figure 6c, the depletion depth is measured from the middle of the NW edge in the perpendicular direction: W_d_ = L/2 − d*. The dotted line calculated with Equation (1) matches the numerical simulations. The situation is different when the depletion depth is taken diagonally from the corner of the NW. Since the effective field at the corners is higher by roughly $\sqrt{2},\;$the depletion depth is accentuated as if the doping in Equation (2) was lower by about 50%. This result is similar to the concept of voltage-doping transformation proposed by Skotnicki et al. [7].

**Figure 7:**
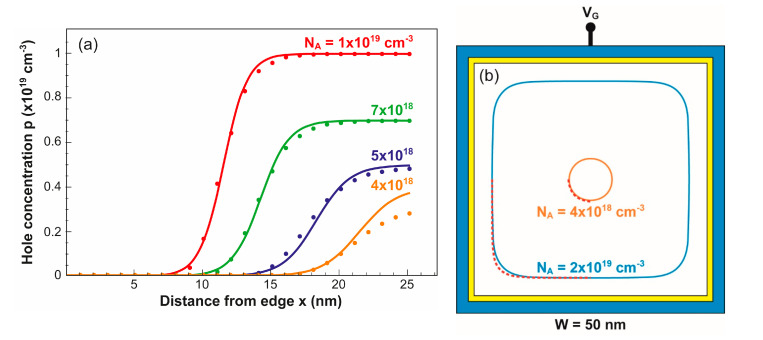
(**a**) Profiles of hole concentration along a horizontal cut from the middle of the NW sidewall to the center. (**b**) Contours of the neutral region produced by numerical simulations (lines) and computed with Equation (3) (dots). V_G_ = 1.5 V.

Figure 7a shows the lateral profile of hole concentration from the left edge to the center of the nanowire. The transition between full depletion and neutral region is sharper as the doping increases due to the reduction of Debye length, as stated in Equation (2). A good agreement with Equation (1) is noted (dotted lines) for high doping. When the size of the conductive region is small (for N_A_ = 4 × 10^18^ cm^−3^ in Figure 7a), the hole concentration is not able to reach the nominal doping level. In other words, the core of the NW is partially neutral or partially depleted, which makes Equation (1) deviate. To recover accuracy, the nominal doping N_A_ needs to be replaced by an effective doping produced by the right section of the gate, which is similar to the case of SOI MOSFETs documented in [6].

The horizontal and vertical effects of depletion can be combined to approximate the 2D distribution of holes. For example, in the bottom left quarter of the NW, we have:

p(x,y) = (N_A_/4) [1 + tanh((x − W_d_)/αL_D_)]·[1 + tanh((y − W_d_)/αL_D_). (3)

The condition p(x,y) = N_A_/2 yields the locus depicting the contour of the conductive NW area:

[1 + tanh((x−W_d_)/αL_D_)]·[1 + tanh((y−W_d_)/αL_D_)] = 2. (4)

Equation (4) is actually an explicit function y(x):

y = αL_D_ tanh^−1^ [(1–tanh((x-W_d_)/αL_D_)) / (1 + tanh((x−W_d_)/αL_D_))] + W_d_. (5)

Figure 7b compares numerically simulated contours with those produced by Equation (5). The contour shape is governed by the doping concentration as seen in Figure 1. A striking aspect is that such a simple, semi-empirical Equation (5) is able to capture the full transition from square shape (N_A_ = 2 × 10^19^ cm^−3^) to circular shape (N_A_ = 4 × 10^18^ cm^−3^, Figure 7b).

The diagonal distance D* from center to contour periphery is obtained by setting x = y in Equation (4):

x = y = (αL_D_/2) ln (√2 +1) + W_d_. (6)

When the diagonal distance D* = √2(W/2 – x) exceeds the horizontal distance d* = W/2 − W_d_, the contour tends to a square shape. In case of a circle, obviously, D* = d*. The doping needed to maintain a quasi-square shape strongly depends on NW size: N_A_ ~ 1/W^2^. In very small nanowires, this doping condition is hard to fulfill and the conductive section becomes naturally round. An interesting limit case is when the circular shape is dominant even at a flat band (Ψ_s_ = 0); this situation is encountered in extremely small NW with a size comparable with the Debye length.

**Figure 8:**
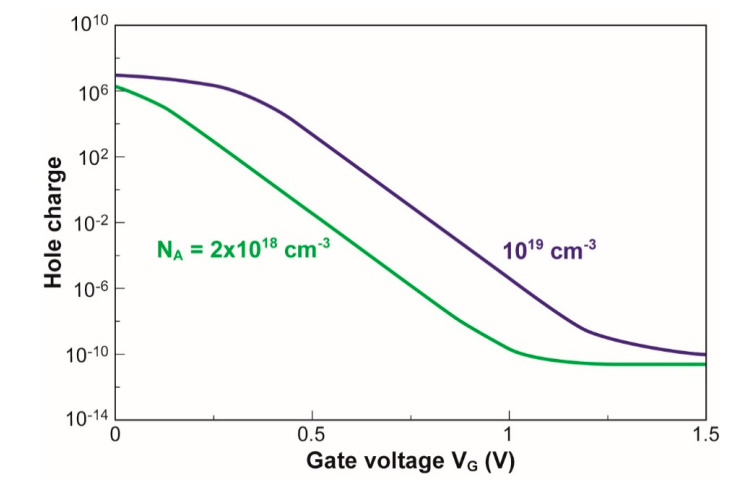
Total hole charge versus gate voltage in two different nanowires (NWs).

The gate biasing modifies not only the concentration of free carriers, as in any MOS device, but also the area and the shape of the conductive channel. However, this triple action is not sufficient to revolutionize the device performance. Figure 8 shows the integral of the hole charge computed in two devices. In the subthreshold region, the charge varies exponentially with gate voltage V_G_. The reciprocal of the slope (subthreshold swing) is constant and corresponds to the thermionic limit of ≈60 mV/decade at room temperature. The concomitant increase in carrier concentration and conductive area cannot break this limit.

## 4. Other Examples

The depletion-mode junctionless GAA NW discussed above served as a simple and generic example illustrating the transition to rounded forms. More complex is the G^4^-FET transistor, where the four sections of the gate are independently biased and feature distinct surface potentials. As a result, the initial dot-like neutral region can grow from any point within the body cross-section, not necessarily from the center. Among many other similar situations, we address two further cases without entering into details.

**Figure 9:**
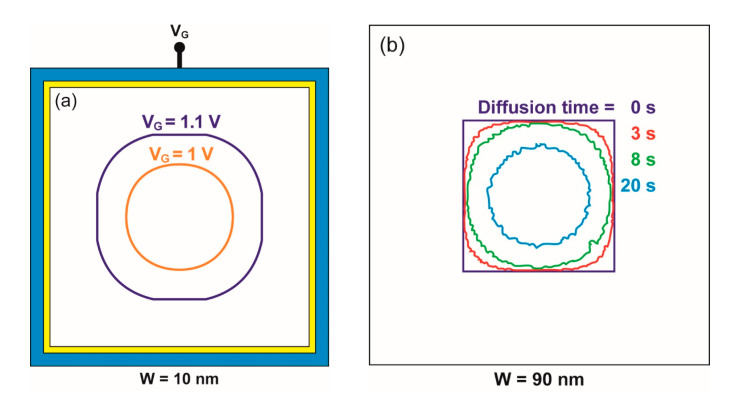
(**a**) Inversion charge contours in subthreshold operation mode of an undoped NW MOSFET. (**b**) Contours showing the evolution of a highly doped core during thermal diffusion in a shell–core NW.

Figure 9a shows the cross-section of an inversion-mode NW GAA MOSFET with undoped body (residual doping N_A_ = 10^15^ cm^−3^) and 10 × 10 nm^2^ size. A positive voltage on the surrounding gate induces an electron inversion region that develops not at the interface but from the center of the NW toward the edges, according to the principle of volume inversion [8]. In the initial stages, the contours of the conductive inversion region are perfectly round. Increasing V_G_ makes the inversion region expand in the whole body at the expense of a contour deformation. Once the threshold voltage is reached, most of the electrons become confined near the interface and corners [9], so reconstructing the original square shape of the NW.

The manufacturing sequence of NW devices could also lead to rounded shapes. During the processing steps, the initially square-shaped piece of semiconductor transforms. It can be via thermal oxidation, isotropic etching, or dopant diffusion. Specifically in Figure 9b, we considered a 100 nm thick core–shell NW. The square core (40 nm) is highly doped, whereas the shell is undoped. At high temperature, dopant diffusion proceeds from the core into the shell, first rounding the corners, and eventually giving rise to a circular core, the area of which keeps shrinking. Finally, the thermal oxidation of the same piece of semiconductor leads to a similar transformation of the square into a circle.

## 5. Conclusions

The conductive region of a NW and even its physical shape can transform from square to circular, depending on gate bias, doping level, and/or technological processes. A criterion for this transition was defined based on systematic simulations. In junctionless GAA NW transistors, the depletion mechanism develops preferentially from the corners, rounding them, and it ultimately achieves a circular shape of the neutral region. Lower doping, a smaller NW cross-section, and higher gate voltage assist this transformation. A quasi-perfect circle is obtained for ≈10 nm diameter of the effective NW region. Since nanodevices tend to become round anyway, circular nanowire grown vertically by epitaxy are well adapted. Empirical relations, able to reproduce the carrier profiles and suitable for compact models, were proposed. These results can be extended to a multitude of nano-size devices, offering a comprehensive root for detailed physical modeling.
